# Challenges in reaching patients with severe mental illness for trials in general practice—a convergent mixed methods study based on the SOFIA pilot trial

**DOI:** 10.1186/s40814-023-01395-y

**Published:** 2023-10-31

**Authors:** Katrine Tranberg, Tina Drud Due, Maarten Rozing, Alexandra Brandt Ryborg Jønsson, Marius Brostrøm Kousgaard, Anne Møller

**Affiliations:** 1 The Section of General Practice and the Research Unit for General Practice, Department of Public Health, University of Copenhagen, Copenhagen, Denmark; 2 The Mental Health Services in the Capital Region of Denmark, Copenhagen, Denmark; 3 Psychiatric Center Copenhagen, Copenhagen, Denmark; 4 The Research Unit for General Practice, Department of Community Medicine, Faculty of Health Sciences, UiT The Arctic University of Norway, Tromsø, Norway; 5 Department of People and Technology, Roskilde University, Roskilde, Denmark

**Keywords:** Trial recruitment, Severe mental illness, Pilot and feasibility studies, General practice, Mixed methods study

## Abstract

**Background:**

Patients with severe mental illness (SMI) die prematurely due to undetected and inadequate treatment of somatic illnesses. The SOFIA pilot study was initiated to mend this gap in health inequity. However, reaching patients with SMI for intervention research has previously proven difficult. This study aimed to investigate the recruitment of patients with SMI for the SOFIA pilot study in 2021.

**Methods:**

We used a mixed-method convergent design. The qualitative material comprised 20 interviews with general practitioners (GPs) and staff, during patient recruitment. The quantitative data consisted of process data on baseline characteristics, GPs reported reasons for excluding a patient, reported reasons for patients declining participation, and registered data from a Danish population of patients with SMI. We used thematic analysis in the qualitative analysis and descriptive statistics for the quantitative analysis. Pillar integration was used for integrating the material.

**Results:**

Our findings show that selection bias occurred in the pilot study. We describe four main themes based on the integrated analysis that highlights selection issues: (1) poor data quality and inconsistency in defining severity definitions troubled identification and verification, (2) protecting the patient and maintaining practice efficiency, (3) being familiar with the patient was important for a successful recruitment, and (4) in hindsight, the GPs questioned whether the target population was reached.

**Conclusions:**

In the light of theories of professions and street-level bureaucracy, we find that the main drivers of the patient selection bias occurring in the SOFIA pilot study were that 1) GPs and staff mended eligibility criteria to protect certain patients and/or to minimize workload and maintain efficiency in the practice 2) the data from the GP record systems and the digital assessment tool to assist recruitment was not optimal. Interventions targeting this patient group should carefully consider the recruitment strategy with a particular focus on professionals’ discretionary practices and information technology pitfalls.

**Trial registration:**

The pilot trial protocol was registered on the 5th of November 2020. The registration number is NCT04618250.

## Key messages

- *What uncertainties existed regarding the feasibility?*
  The recruitment strategy was not previously assessed. It was unclear whether it would be possible to reach the target population of patients with SMI.
- *What are the key feasibility findings?*
  Selection bias occurred since (1) the identification of patients in the record systems was troublesome and the data quality was low, and (2) GPs and staff selected patients based on their perceptions of who would benefit from participating and to minimize additional workload in the practice.
- *What are the implications of the feasibility findings for the design of the main study?*
  The recruitment strategy ought to be altered and reassessed. The use of record data and assessment tools for recruitment purposes in general practice settings should be performed with precaution.

## Introduction

“I most likely included the patients for whom I thought it was possible to take part in a research project. But in reality, it would have made more sense to include the ones where I didn’t think it was possible…”. These are the words of a general practitioner (GP, male) discussing the recruitment of patients with severe mental illness (SMI) during a reflection seminar after having participated in the SOFIA pilot study. The study examined the implementation of an intervention purported to reduce mortality and improve the quality of life in patients with SMI [1]. Previous literature [2] has suggested that recruiting patients with SMI in intervention research is a challenge and that the use of clinicians as recruiters in research might introduce selection issues [3]. In this work, we intend to gain a deeper understanding of using GPs and staff to recruit patients with SMI for intervention research based on results from the SOFIA pilot study. 

Patients with SMI die prematurely compared to the general population [4, 5]. New interventions, treatments, or healthcare system architecture are needed to mend this gap in health inequity [5]. The cause of the disparity in mortality is primarily due to somatic illnesses, notably cardiovascular disease [6], which is often not timely recognized or treated adequately [5]. General practice has been suggested as a setting for implementing novel interventions to address this issue [7], and continuity of care with a GP has been shown to have a positive impact on acute hospitalization, use of out-of-hours care, and mortality [8]. However, investigating the effects of interventions to reduce mortality in patients with SMI not only requires a structured approach to intervention delivery, it also requires that the intervention manages to reach the target population—which refers to the level of contact or participation of the intended audience of a particular initiative, program, or intervention [9]. 

Studies have identified several barriers related to the unsuccessful recruitment of patients with SMI in clinical trials [10]. These challenges include practicalities such as weather, transportation costs, and location. However, perceived stress regarding family or other close relations, the state of the mental illness, being physically low-functioning as well as disagreeing with the SMI diagnosis have also been found to be highly associated with successful recruitment and retention [10]. Moreover, many patients with SMI have experienced being stigmatized and discriminated against when encountering the healthcare system [11].

Using GPs as recruiters in interventions has been investigated previously. Guillemin et al. [12] found that GPs involved in research often find it hard to navigate the dual role of being a care provider and researcher. This is described as an ethical dilemma for the clinician; on the one hand, the GP needs to follow the systematic approach demanded by the trial and on the other being responsible for the patient’s welfare [2, 12, 13]. Also, barriers like forgetting to invite patients to participate due to heavy workloads, or feeling uncomfortable or embarrassed by inviting the patient, have been highlighted by GPs to influence recruitment in trials [2, 3, 14]. 

A range of different recruitment strategies have been tested and evaluated in research targeting patients with SMI, but comparing the effectiveness and impact of different strategies has proved to be difficult [15]. In this paper, we explore the process and outcome of recruiting and reaching patients with SMI for the SOFIA pilot study through:

1. Investigating selection bias by differences in baseline characteristics between recruited and non-recruited patients for the SOFIA pilot study and comparing these groups with a general register-based population of patients with SMI.
2. Exploring how patient recruitment was performed by general practitioners and general practice staff and the reasoning behind the choices and actions taken in the recruitment process.

To our knowledge, no previous papers have addressed the recruitment of patients with SMI from a mixed methods perspective in a general practice setting. The integration of qualitative and quantitative methods makes it possible to extend the exploration of challenges in the recruitment process and examine how, as well as why, recruitment barriers of patients with SMI exist in intervention research.

## Methods

The data collection presented in the article is based on a mixed-methods convergent design [16]. The convergent aspect entails qualitative and quantitative data being collected simultaneously throughout the recruitment process in the SOFIA pilot study.

### The SOFIA intervention

The setting was General Practice in Denmark. Access to healthcare services in general practice is free of charge to all Danish citizens, and the GP acts as a gatekeeper to in- and outpatient hospital care and other specialized services [17]. The SOFIA intervention elements were developed through a co-design phase and feasibility testing [18]. The intervention comprised three components: (1) an extended consultation, following the developed SOFIA scheme for conducting a consultation; (2) a course for GPs and staff introducing the intervention activities and the evidence supporting the study; and (3) a handbook containing relevant referral options in the municipality and regions [1].

The SOFIA pilot study was conducted between October 2020 and October 2021 in Region Zealand and the Capital Region of Denmark as a cluster-randomized controlled trial. During autumn 2020, 12 general practices were recruited to participate in the study. We ended up including nine practices since three practices dropped out before finishing patient recruitment. The practices were allocated in a 2:1 ratio resulting in six practices in the intervention group and three practices in the control group. The control group was instructed to provide care as usual, which in Denmark equals free access to services in general practice during standard and out-of-office hours.

### Patient recruitment and eligibility criteria

The patient recruitment strategy included four steps. First, general practices were instructed to extract lists from their record systems of patients fulfilling diagnostic or medication prescription criteria for inclusion. Secondly, these lists were securely submitted to the trial management team, where an algorithm randomly selected 15 patients corresponding to the diagnostic criteria for SMI set by the research group as described below (in total 45 patients per practice). Thirdly, the general practitioners were instructed to verify the SMI diagnoses and assess the eligibility of the patients. Finally, practices contacted eligible patients, until 6–15 patients were included, with at least two patients from each diagnostic group (severe depression, bipolar disorder, and psychotic disorder). Information material for recruitment purposes was developed and provided by the trial management team to assist the practices during the recruitment process [1].

**Fig. 1:**
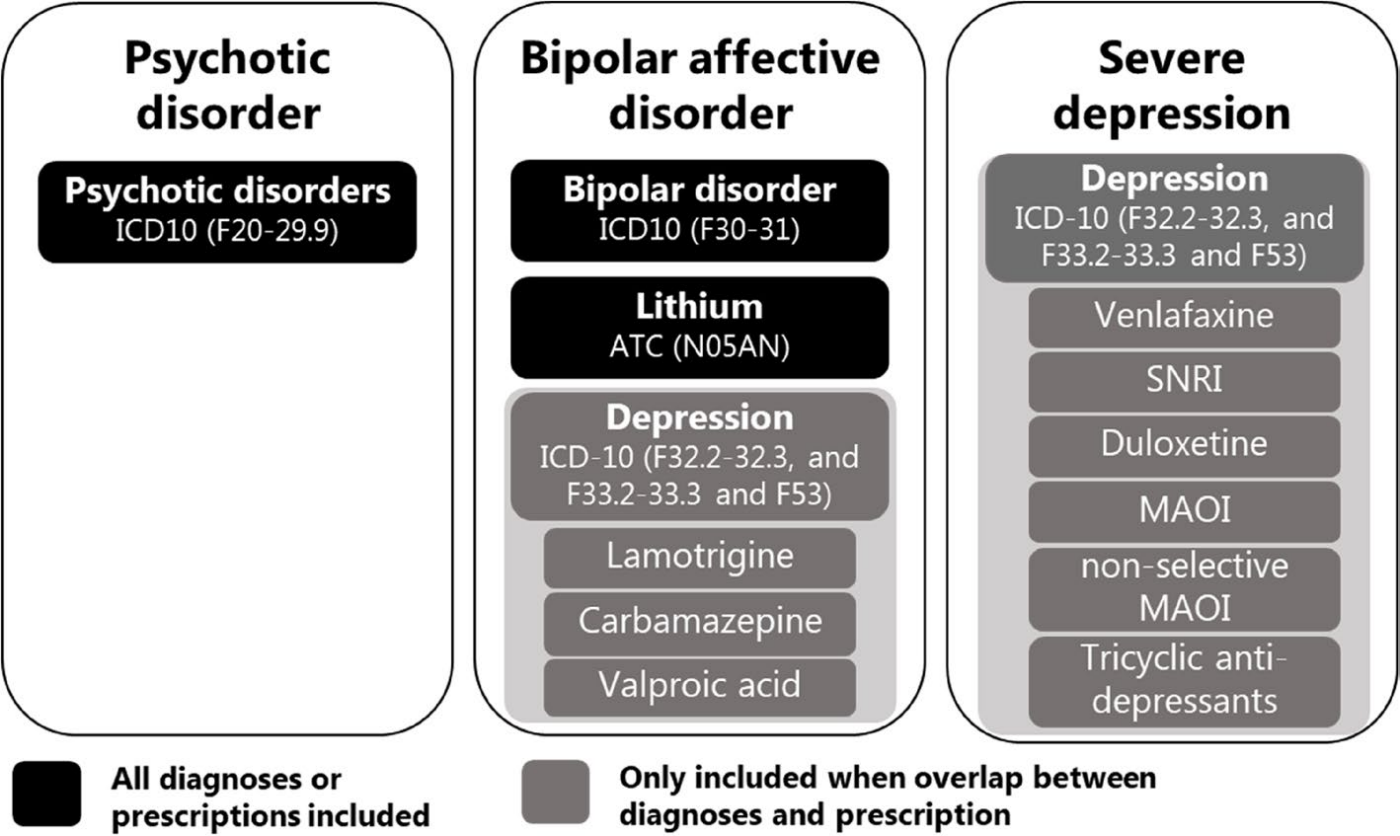
Inclusion criteria of patients with severe mental illness in the SOFIA pilot study

Patients were eligible to participate if they were above 18 years and were diagnosed with a psychotic disorder, a bipolar mood disorder, or a severe unipolar depressive disorder. The exact definitions of the inclusion diagnoses are shown in Fig. 1. Patients were excluded from the study if they had been subjected to involuntary commitment in the Danish Mental Health Care Act (Psykiatriloven); were registered with a dementia diagnosis, organic psycho-syndrome, or other neurological diseases; received end-of-life care; were non-Danish speakers; or if the psychiatric diagnosis appeared to be incorrect or outdated. To minimize the risk of selection bias, GPs were only allowed to exclude patients for other reasons than the exclusion criteria if the assessment was conferred with the trial management team.

### Quantitative data

#### Process data on patient recruitment

GPs and staff were instructed to collect and assess information on patient recruitment in REDCap [19]. This included information on diagnosis verification, which patients were excluded and why, baseline characteristics, who contacted the patient, and the stated reason for not participating in case the patient declined.

#### Register sample of patients with SMI

We used register data from The Danish Psychiatric Central Research Register [20] and the National Patient registry [21] to compare SMI diagnosis and sociodemographic characteristics between the included patients and eligible patients for the SOFIA pilot study with a general sample of patients with SMI. Patients were included in the register sample if they fulfilled the following criteria:

- Registered with a diagnosis in the psychiatric register in 2018 with either psychotic disorder, bipolar disorder, or severe depression (following the ICD-10 criteria displayed in Fig. 1—excluding medication). The latest diagnosis was used if patients were registered with more than one SMI diagnosis in 2018
- 18 years or older
- Not registered with dementia, organic psycho-syndrome, or any other neurological diagnosis equal to ICD10 codes F0 (all) and G(all) up to five years before enrolment.
- Living in Region Zealand or the Capital Region of Denmark at study enrollment

Furthermore, we computed a randomly selected sub-sample from the register population with an identical distribution of SMI diagnoses as the included patients in the SOFIA pilot study called “importance sampling” (see the section on data analysis for further details).

#### Patient baseline characteristics

Baseline information was obtained from registers provided by Statistics Denmark during 2019–2020. The data consisted of information on age, sex, highest attained education (primary school/not registered, secondary education, higher education), civil status (married/partner, single), employment status (unemployed, employed, pensioner), and income (quartiles).

To assess the need for services, we used diagnoses with somatic comorbidities and former use of GP services as a proxy. Data on comorbidities were registered in REDCap for patients in the SOFIA study and were only available in the National Patient Registry [21] for the register sample up till 2018. We used a count number of comorbidities per individual three years before study entry based on the Quality and Outcomes Framework (QOF) [22] divided into three categories (0, 1, ≥ 2). We used the QOF since this framework has previously been highlighted as a useful predictor of costs and mortality in primary health care [23, 24]. Previous use of GP services was defined as the number of GP contacts in 2020—the year before the study entry.

### Qualitative data—interviews and discussions with general practitioners and practice staff

The qualitative data consisted of 20 interviews with GPs and staff conducted during the recruitment process. We also included discussions from a reflection seminar hosted at the end of the pilot study. The interview data was collected between January 1, 2021, and March 1, 2022. The interviews were performed by KT in the nine practices included in the pilot study and in two of the practices that were excluded before randomization. The remaining practice (practice 12) declined participation due to heavy workloads. Interview participants were chosen based on their role in the recruitment process. In each practice, a GP (*n*=11) who undertook the patient recruitment was interviewed, and if a staff member assisted (*n*=9), they were interviewed as well. Professionals were invited to participate by phone or email. The interview guide (Appendix) was structured to follow the process of patient recruitment. Each element of the recruitment process was provided with a question and follow-up questions were formulated to assist the interviewer. The interview guide was thoroughly discussed between the researchers (KT, TD, and AJ), and smaller alterations to clarify the questions were included iteratively during the interview phase. The interviews lasted from 30 to 75 min. Because of the COVID-19 pandemic, 10 interviews were conducted by phone and 10 interviews were conducted in the practice. An overview of the interview participants is shown in table 1. 

The reflection seminar was hosted 2 weeks after finalizing the pilot study in October 2021. GPs from five intervention practices and two control practices participated (Table 1); however, GPs from all participating practices were invited. The GPs were introduced to the preliminary findings from the pilot study and discussed the implementation in practice based on their experiences. Discussions were recorded and facilitated by AJ, AM, and KT.

**Table 1:** Characteristics of interview participants

Practice	Allocation	Region	Practice type (*n* GPs)	Practice size (*n* patients/*n* GPs)	Participant in interview (sex)	Participant in reflection seminar (sex)
1	Intervention	Capital	Solo (*n* = 1)	1900	GP (m)	GP (m)
2	Intervention	Capital	Solo (*n* = 1)	850	GP (f)	
					Practice manager (f)	
3	Intervention	Capital	Group (*n* = 4)	1600	GP (m)	GP (m)
					Nurse (f)	GP (m)
4	Intervention	Capital	Group (*n* = 2)	1625	GP (m)	GP (m)
					Secretary (f)	GP (f)
5	Intervention	Zealand	Group (*n* = 3)	1567	GP (f)	GP (f)
					Secretary (f)	
6	Intervention	Zealand	Solo (*n* = 1)	1950	GP (f)	
					Nurse (f)	
7	Control	Capital	Group (*n* = 3)	1667	GP (f)	GP (f)
8	Control	Capital	Group (*n* = 4)	1600	GP (m)	GP (m)
					Nurse (f)	
9	Control	Zealand	Group (*n* = 4)	1825	GP (f)	GP (f)
					Nurse (f)	
10	Excluded	Zealand	Group (*n* = 4)	Missing data	GP (m)	
					GP (f)^a^	
					Secretary (f)^a^	
11	Excluded	Zealand	Group (*n* = 3)	Missing data	GP (f)	
12	Excluded	Zealand	Group (*n* = 3)	Missing data	None	

^a^GP and secretary interviewed together; In one of the interviews performed in practice 10, a GP and a secretary wished to be interviewed together

The participants were provided information about the study verbally and in text and gave their signed informed consent prior to the interviews and reflection seminar. Participants were informed about their right to withdraw from the study at any time.

### Data analysis

The quantitative data were analyzed in SAS version 9.4. We used a chi-squared test for categorical variables to investigate significant statistical differences between exposure groups with a chosen significance level of < 0.05. A random sample from the register population was generated Using the “Survey Select” procedure. The distribution of SMI diagnoses in this random sample was standardized with the included SOFIA study population as a reference. Findings were categorized into meaning units by comparing findings on differences and similarities in process data, SMI diagnosis distributions, and differences in characteristics between the SOFIA and register-based populations.

The qualitative analysis followed a thematic approach as described by Lochmiller [25]. All interviews, as well as discussions from the reflection seminar related to patient recruitment, were transcribed using a pre-developed transcription guide focusing on the content of the interviews. Subsequently, KT read the material thoroughly and developed the first draft of the initial codebook. KT, TD, AM, and MB then applied the codebook to two interviews and compared and revised it during several rounds of discussions before reaching an agreement on the final version of the codebook. KT coded the remainder of the interviews and discussions from the reflection seminar, using NVivo (2020). Facilitated by a discussion in the author group, we began organizing the codes into categories of meaning units.

The integrative analysis of qualitative and qualitative data was based on the Pillar integration process for producing a joint display as described by Johnson et al. [26]. The findings, codes, and categories created from the quantitative and qualitative data were initially listed and matched by KT in preparation for developing the integrative joint display visualizing the analysis and findings. Categories and codes were thoroughly reviewed and checked for similarities and discrepancies in the material. Finally, from the listing, matching, and checking process, the author group identified themes across the categories by comparing and contrasting findings—building the center pillar of the joint display.

## Results and findings

### The SOFIA recruitment process in numbers

**Fig. 2:**
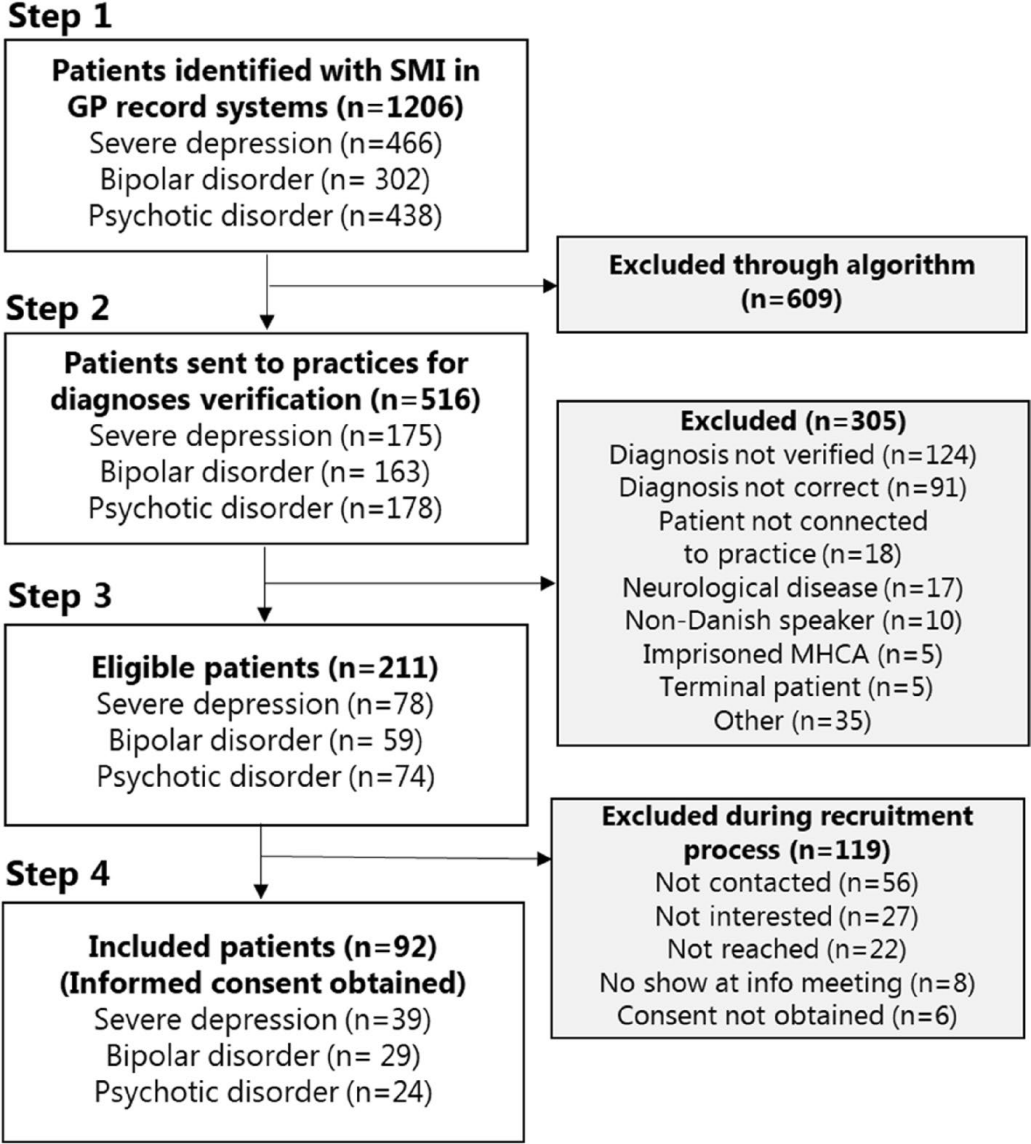
Flow chart of the patient recruitment process presenting an overview of reasons for exclusion

An overview of the patient recruitment process is presented in Fig. 2. Initially, patient data were extracted from the record systems. An algorithm used data from 1206 patients identified as having SMI based on the predetermined criteria (Fig. 1), and 516 patients were randomly selected and returned to the practices where the SMI diagnosis and eligibility criteria were verified by the GP. During the verification process, 305 patients were excluded. The majority of these patients (47.8%) met the exclusion criteria (diagnosis not correct, deceased or relocated, neurological disease, non-danish speaker, imprisoned, and terminal patient). However, 124 of the excluded patients (40.7%) never had their diagnosis or eligibility criteria verified and 35 patients (11.5%) were excluded due to other reasons. Although professionals were instructed to confer with the trial management team if they considered excluding patients due to other reasons than the predetermined exclusion criteria, none reached out. Moving on in the recruitment process, 211 patients were found eligible. Of the eligible patients, some were not interested in participating (*n* = 27), some could not be reached (*n* = 22), and 56 of the eligible patients were never contacted (26.5%). In total 92, patients were included in the SOFIA pilot study.

### Characteristics of included, eligible, and register sample patients

Table 2 shows the characteristics and comparisons between the following four groups: (1) Included patients: the 92 included patients in the SOFIA study, who signed the informed consent for participation; (2) Eligible patients: the 119 patients who fulfilled the inclusion criteria but were not included in the study; (3) register-sample: a register-population of 17,229 patients registered with SMI in Region Zealand and the Capital Region of Denmark in 2018; and (4) importance sampling: the randomly selected sub-sample with an equal distribution SMI diagnoses as the included patients. Table 3 shows the associated *p* values.

**Table 2:** Baseline characteristics of included, eligible, and registered sample patients

	Included patients		Eligible patients		Register sample		Importance sampling	
	*N*	(%)	*N*	(%)	*N*	(%)	*N*	(%)
Total	92		119		17,229		3509	
Psychiatic diagnosis^†‽^								
Severe depression	39	42.4	39	32.8	1488	8.6	1488	42.4
Bipolar disorder	29	31.5	30	25.2	3182	18.5	1106	31.5
Psychotic disorder	24	26.1	50	42.0	12,559	72.9	915	26.1
Sex^†‽^								
Male	31	33.7	52	43.7	8,699	50.5	1460	41.6
Female	61	66.3	67	56.3	8,530	49.5	2049	58.4
Age^†‡‽^								
18–29	12	13.0	18	15.1	4369	25.4	715	20.4
30–39	17	18.5	21	17.7	3505	20.3	635	18.1
40–49	21	22.8	19	16.0	3128	18.2	592	16.9
50–59	16	17.4	27	22.7	2998	17.4	663	18.9
60–69	20	21.7	23	19.3	1883	10.9	450	12.8
70 = <	6	6.5	11	9.2	1346	7.8	454	12.9
Mean (SD)	48 (15.3)		50 (16.3)		44 (16.5)		47 (17.8)	
Contacts to GP 2020^†‽^								
0–5	24	26.1	42	35.3	7694	44.7	1369	39.0
6–10	26	28.3	22	18.5	3698	21.5	812	23.1
11–15	18	19.6	22	18.5	2194	12.7	496	14.1
16–20	6	6.5	12	10.1	1328	7.7	315	9.0
20 <	18	19.6	21	17.7	2315	13.4	517	14.7
Substance use disorder^‽^								
No	82	89.1	107	89.9	15,644	90.8	3242	92.4
Yes	10	10.9	12	10.1	1585	9.2	267	7.6
N. Somatic comorbidities^*†‡‽^								
0	35	38.0	67	56.3	14,427	83.7	2827	80.6
1	31	33.7	32	26.9	2073	12.0	486	13.9
> 2	26	28.3	20	16.8	729	4.2	196	5.6
Income in 2020^*†‽^								
< 150.000 kr	25	27.2	41	34.5	6343	36.8	1080	30.8
150.001–225.000 kr	29	31.5	48	40.3	7036	40.8	1323	37.7
225.001–300.000 kr	16	17.4	20	16.8	2391	13.9	594	16.9
> 300.000 kr	22	23.9	10	8.4	1459	8.5	512	14.6
Highest attained education 2020^*†‽^								
Primary school/not registered	23	25.0	60	50.4	8392	48.7	1279	36.5
Secondary school	42	45.7	35	29.4	5683	33.0	1323	37.7
Higher education	27	29.4	24	20.2	3154	18.3	907	25.9
Working status^†‽^								
Working	23	25.0	19	16.0	2183	12.7	696	19.8
Out of work	54	58.7	82	68.9	13,249	76.9	2247	64.0
Pensioners	15	16.3	18	15.1	1797	10.4	566	16.1
Civil status^*†‽^								
Single	58	63.0	93	78.2	13,141	76.3	2315	66.0
Married/partner	34	37.0	26	21.9	4088	23.7	1194	34.0

Significance level < 0.05

^*^Significant *p* value between included and eligible patients

^†^Significant *p* value between included patients and registered sample

^‡^Significant *p* value between included patients and the importance of sampling

^‽^Significant *p* value between registered sample and the importance of sampling

**Table 3:** *p* values from chi-squared tests

	Included patients vs. eligible patients	Included patients vs. register sample	Included patients vs. importance sampling	Register sample vs. importance sampling
Psychiatic diagnosis	0.05	< 0.001^*^	1.00	< 0.001^*^
Sex	0.14	0.001^*^	0.13	< 0.001^*^
Age	0.73	0.01^*^	0.03^*^	< 0.001^*^
Contacts to GP 2020	0.34	0.01^*^	0.07	< 0.001^*^
Substance use disorder	0.85	0.58	0.25	< 0.001^*^
N. Somatic comorbidities	0.02^*^	< 0.001^*^	< 0.001^*^	< 0.001^*^
Income in 2020	0.02^*^	< 0.001^*^	0.09	< 0.001^*^
Highest attained education 2020	< 0.001^*^	< 0.001^*^	0.08	< 0.001^*^
Working status	0.22	< 0.001^*^	0.45	< 0.001^*^
Civil status	0.04^*^	0.003^*^	0.56	< 0.001^*^

^*^Significant based on *a* < 0.05 significance level

The difference in SMI diagnoses distribution between the included patients and eligible patients was borderline statistically significant. A larger percentage of included patients had severe depression compared to psychotic disorders in the eligible patients. Included patients also differed significantly from eligible patients by having more somatic comorbidities, a higher income, a higher education status, and being more likely to live with a partner. Furthermore, included patients had a different GP contact pattern. The eligible patients contacted the GP more frequently, however not significant.

Apart from substance use disorder (SUD), all baseline characteristics of the register sample were significantly different from those of the included patients. The distribution of SMI diagnosis is particularly noticeable since it seems inverted in the register sample, with the largest proportion constituting patients diagnosed with psychotic disorder and only 8.6% with severe depression.

Finally, the importance sampling differed in some of the baseline characteristics. Patients in the importance sampling group had significantly fewer somatic comorbidities and a larger (but not significant) proportion of patients with 0–5 GP contacts in the year 2020 (39%) compared to the included patients (26.1%).

### Merging the quantitative and the qualitative findings from the patient recruitment process

**Fig. 3:**
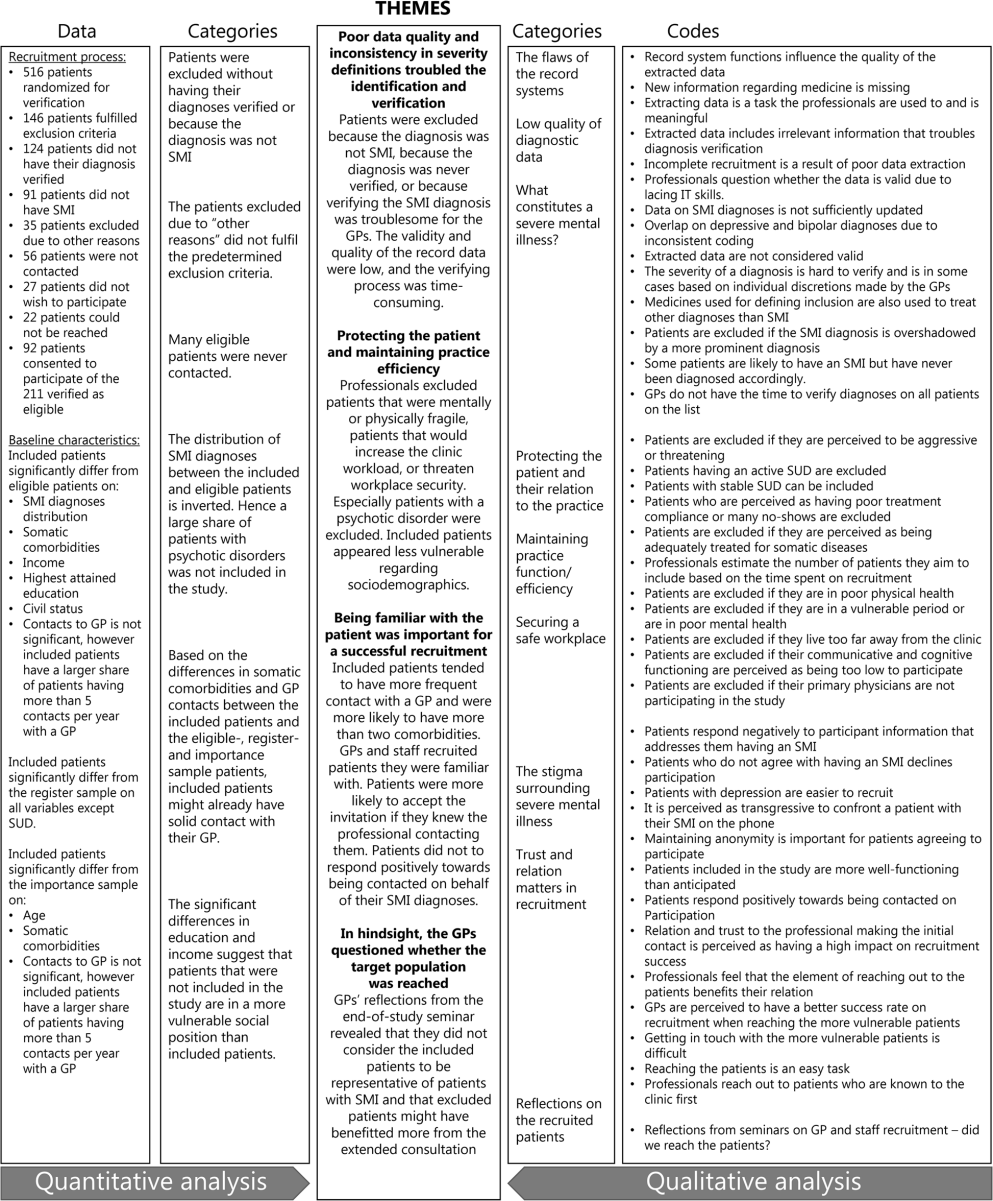
Joint display of mixed methods analysis

In the qualitative analysis, we merged the initial codes into nine categories describing how the recruitment process was enacted and ascribed meaning to by the GPs and staff. The categories were merged with the quantitative findings presented in four overlying themes below and visualized in a joint display in Fig. 3.

#### Poor data quality and inconsistency in severity definitions troubled the identification and verification

Identifying patients with SMI by using data from the practice record systems and the REDCap assessment tool was a difficult task to perform, and the professionals experienced several challenges in this aspect of the recruitment process. First, there were varying opinions about using data from practice record systems for research purposes. A few GPs and staff members were comfortable performing data extraction and found the data valid and useful for identifying patients.

“.. we often extract data to create an overview of our patients with chronic disease i.e. COPD (..) It is just a tool from our everyday practice. Not that we do it every day, but often.” (Interview, Nurse, practice 9)

Others strongly believed that the record systems did not provide the tools necessary to extract valid data and therefore found the data misleading. Some GPs explained that patients, whom they knew to have a valid SMI diagnosis, were not included in the extracted data. This was described by a GP who explained that they identified very few patients with SMI in her practice.

“Something must have happened, because we did not identify the right patients. (..) We know that some of our patients have these diagnoses, and have had them for many years and furthermore have the correct prescriptions.. We don’t really get it..” (Interview, GP, practice 11)

Another aspect that troubled the practices in identifying the patients was the quality of the diagnostic codes entered into the record systems. Information regarding the severity or the state of the mental illness was not always up to date, and it was not necessarily clear to the GPs, which diagnosis was the most prominent or whether it could be perceived as severe. It was a time-consuming process that required a thorough search of the patient’s records to gain information on when, by whom, and on what indication the diagnosis was given.

“.. In many cases I had to change the diagnosis that was originally registered (in the system) and I had to overlook a pretty comprehensive data material (..) because I had to find out who diagnosed the patient and then answer all of the questions related to inclusion, right?” (interview, GP, practice 3)

Consequently, several GPs explained that to limit time consumption, they initially verified patients whom they knew to have a valid SMI diagnosis. Moreover, the medication prescriptions were not always sufficient to verify the severity. In some cases, GPs had to use their perception of what a “severe” mental illness entailed. As an example of this, a GP explained that the prescription of medications sometimes overlapped with diagnoses not relevant for study inclusion:

“A lot of patients, who do not have a bipolar disorder, are prescribed mood stabilizers... So, some of the patients that were chosen (added to the list based on data from the record system), actually had epilepsy because the medication is the same right? And some of them were just depressed … so it was a very heterogeneous group of patients, right?”. (Interview, GP, practice 6)

One GP also described that the information in the record systems (including diagnoses) should perhaps be considered as a tool for the individual GP to assist their daily work, not as a valid diagnostic data source to use for research purposes:

“Well… The dream is to just press a button and then get a correct and complete list of data. But often when we are dealing with record data, half of it is pure garbage... You have to verify everything to make sure it is correct, and that really makes the research part of it extremely challenging.. “ (Interview, GP, practice 1)

In relation to the quantitative data, the perceived problems of record data quality illustrate that patient selection could occur when extracting data from the system (step 1), as well as when verifying the diagnosis (step 2), which may explain the large share of patients whose diagnosis appeared to be incorrect as illustrated in the flowchart (Fig. 2). Since the diagnosis verification was considered time-consuming to the GPs, many patients never had their diagnosis verified, and patients who were known to have a SMI diagnosis were included first. The differences in the distribution of SMI diagnoses between the included, eligible, and register sample patients could be related to both identification and verification issues as well as to GPs finding the severity assessment of the mental illness hard to perform.

#### Protecting the patient and maintaining practice efficiency

The professionals explained that in some instances, they chose not to include patients to either protect the patient or to avoid adding to the already heavy workload.

“Of course our selection shouldn’t be too biased (..) but anyway we did have some thoughts or considerations about what we could expect from the patients, and if they were able to participate or not.” (Interview, GP, practice 9)

In these situations, GPs and staff prioritized not to verify the diagnosis, exclude the patient for other reasons than the exclusion criteria, or not contact patients whom they believed would be either too troublesome to enroll to participate in the study or who would not benefit from it. If the patient was not perceived to benefit from the intervention, it was because they were undergoing treatment elsewhere, residing far from the practice, or were considered too physically or mentally frail to participate in the study. In this regard, one GP described a patient, who did fulfill the inclusion criteria, but whom she did not think would benefit from the study:

“.. One of the patients that come to mind, she has schizophrenia and then she used to have a massive alcohol consumption, so mentally she is just… I mean she wouldn’t be able to participate... She phones us anyway on a daily basis. I don’t think that... It would be really heavy on us to include her, and she probably wouldn’t profit from it. She wouldn’t be able to understand the purpose...” (Interview, GP, practice 7).

In other cases, the professionals did not include patients, if they feared it would jeopardize the relationship they had already established with the patient. They also considered whether to include patients who were known to have many no-shows because it would be a struggle getting these patients to complete the study. A few practices also excluded patients, who potentially could threaten workplace security. One GP described that she:

“.. Tried to be as objective as possible and just recruit based on the criteria given. But… Sometimes the patients are not really suited to participate because they are dangerous, right? … That wouldn’t be beneficial for anyone. So… They can’t be too unstable in their schizophrenia, if they start to carry a weapon and you begin to be insecure having them in the practice, then they wouldn’t be smart to include, I think.” (Interview, GP, practice 11)

These examples illustrate that the professionals excluded patients for other reasons than the exclusion criteria determined in the study. Concerning the flowchart (Fig. 2), this type of patient selection is related to several points in the process: (1) patients not having their diagnosis verified, (2) patients being excluded due to “other reasons,” or (3) patients not being contacted (step 2 + 3, Fig. 2). Moreover, not including patients perceived as frail can be detected in the differences in the distribution of SMI diagnoses and socio-economic characteristics shown in Table 1 between the included and eligible patients. 

#### Being familiar with the patient was important for a successful recruitment

In the professionals’ perspective, patients who were known in the practice were perceived as more likely to agree to participate in the study and hence result in successful recruitment.

“It is the patients that know me, I mean where we have a good relationship and they see me regularly who would definitely be easier to recruit than the patients who attends appointments at my coworker, in the psychiatry or elsewhere, right?” (interview, GP, practice 7)

This meant that patients with an already established contact were included and contacted first. Moreover, the professionals imagined that patients would react more positively to a study invitation if they knew the GP, secretary, or nurse contacting them. Thus phone calls were delegated to a person having an established relationship with the patient:

“So if I (the patient’s primary GP) call and ask them about something then they often go like *Well of course we would like to participate because what you represent is something we agree with*. .. So I know that if I call them myself then they accept the invitation, because it makes sense for them ... *haha* sorry, this is really cheesy, but it is just easier .. they think it is a good idea if I am in on it. That’s actually all there is to it.” (Interview, GP, practice 6)

Many professionals reported that patients responded positively when invited to participate in the study and that some patients even expressed gratitude when they received the invitation. A few professionals, however, had a different experience. A secretary, who had just started working in the practice, and thus did not yet know the patients, explained that she was unsuccessful in recruiting patients in the beginning. She used the information guide template for contacting patients provided by the trial management team, which stated that the patient was invited based on their SMI diagnosis and in her experience, this was a barrier to the recruitment:

“It didn’t matter whom I contacted, no one was willing to participate … I quickly discovered that patients with a depression, were not as sensitive to what I told them from the contact instructions (provided by the trial management team), whereas if I mentioned *mental illness* to patients with other diagnoses then there was.. they were not able to grasp this, and almost completely shut down and didn’t want to hear any more about the study.” (Interview, Secretary, practice 4)

To mend this barrier, the secretary and GP decided to change their recruitment strategy. This entailed that the GP contacted patients perceived as more frail without mentioning the mental illness when talking to the patients. 

Looking at the recruitment process (Fig. 2), prioritizing the inclusion of patients known in the practice was performed when professionals verified SMI diagnosis and when deciding which patients to contact first. In total, 27 patients, out of the 155 that were contacted, actively declined the invitation. This adopted recruitment strategy likely influenced the low rate of patients declining. Moreover, the baseline characteristics showed that included patients tended to have more yearly contacts with their GP than patients from the eligible, register, and importance sample groups.

#### In hindsight, the GPs questioned whether the target population was reached

At the end of the pilot study, the trial management team hosted a seminar where the GPs were asked to reflect on the preliminary results from the pilot trial. When introduced to the data material from the recruitment process, it was discussed to which degree the target population was reached. Here, issues related to identifying patients with SMI in the record systems, that patients, who were known with an SMI diagnosis, were not on the lists, and that the recruitment process was too time-consuming, came up again. The GPs suggested that future recruitment should not be based on data from the record systems, but instead, GPs should handpick the patients they considered would benefit from participating. However, the seminar also brought forward a discussion of the GPs’ selection of patients in the study:

- GP, practice 8: “Some of the patients that were not included are probably not suited to be a part of a research project but they might have the same benefit from this intervention.”
- GP, practice 3: “.. maybe benefit even more ..”
- GP, practice 8: “.. yes benefit even more from this than the ones included.”

The GPs did not consider the patients included in the SOFIA pilot study to represent the target population. Included patients were generally perceived as less complex than other patients with SMI. However, the GPs were unsure, if the patients they did not include in the study would be able to participate in a trial, which was their main reason for not including them. 

## Discussion

In this mixed methods study, we investigated the recruitment of patients with severe mental illness (SMI) performed by general practitioners (GPs) and staff in the SOFIA pilot study. Through the integration of quantitative and qualitative findings, we have shown that GPs and staff influenced the recruitment process and introduced selection bias in several steps of the recruitment process; when identifying the patients in the record systems, when verifying the SMI diagnosis, when determining eligibility, and when deciding which patients to contact. The selections performed by professionals were affected by their concerns about poor data quality in the record systems, their perceptions of which patients they believed would benefit from participating in the study, and their considerations of avoiding additional work in the practice. The patient selection had implications for the representativeness of the SOFIA population and thus could bias the results of the pilot trial. Included patients differed from eligible patients, and register sample patients regarding diagnosis distribution, socioeconomic factors, and need-related factors.

### General practitioners and staff: professionalism and street-level bureaucracy

To develop our understanding of the reasoning and actions taken by the GPs and staff when selecting patients with SMI for the SOFIA pilot study, we will discuss the findings in the light of previous literature and by employing concepts from theories of professions and Michael Lipsky’s theory of street-level bureaucracy [27, 28]. For this paper, we view professionalism from two perspectives; the functionalist approach and the neo-weberian approach (or power-oriented approach). The first approach considers professions as altruistic institutions serving societal needs that are concerned with professional norms and ethics and seeks to understand and describe distinctive characteristics of professions [27]. The latter approach emphasizes that professions and professionals are also self-interested actors that use their knowledge and ethics to negotiate, obtain, and maintain status, autonomy, and financial gains in the workplace and society by using professional knowledge and ethics to strengthen their benefits and status [27]. According to Harrits (2019), the SLB theory can be considered to be nested within the neo-Weberian approach [27]. Street-level bureaucrats (SLBs) are frontline workers in the public system who provide services to clients. In a context of finite resources, complex problems, and multiple objectives, they are under constant pressure to enact policy into practice and to meet the demands of many clients with individual needs and requests. To cope with the challenges, SLBs use their discretion to alter and manage their workload by controlling access and demand [28]. Although GPs and staff are not introduced as SLBs in Lipsky’s initial work, the theory has been suggested to “develop (an) understanding of GPs’ behaviours towards guidelines and targets and how these affect patient care.” ([29],p.377). 

#### Professionals protect the pratice and the patient when assessing patient eligibility

Similar to our findings, other papers have suggested that professionals performing recruitment in trials apply their own definitions of patient eligibility. In the functionalist approach, the underlying explanation has been presented as an ethical matter, where the clinicians exercise paternalism in selecting eligible patients for recruitment in trials, applying their perceptions of the patient’s needs in the eligibility assessment [2, 12, 13]. Guillemin et al. suggest that GPs employ gatekeeping practices at the level of the individual patient as “.. a response to the ethical ambiguities generated by their dual roles as a care provider and researcher.” (12],p.103). Thus, the reasoning for selecting patients is a matter of caring for the patients and tending to their needs. However, our findings also suggest that GPs and staff mended eligibility criteria for reasons related to the everyday functioning of the practice. GPs and staff chose not to invite patients with many no-shows, and patients perceived as a heavy burden to include based on their previous contact pattern or lack of communicative or cognitive functioning. This underlines that the professionals exercised discretion not only to protect the patient but also to maintain practice function and efficiency. This behavior is in line with SLB theory where street-level bureaucrats tend to focus on clients that are easier to manage and/or more likely to benefit from services [28]. The selection of patients can be viewed as a means to regulate workflows and prioritize scarce resources in the busy context of general practice. 

In addition to revealing that GPs and staff used discretion in determining patient eligibility, our analysis showed that patients who were familiar in their practice were prioritized when invited to join the study. Previous studies have shown that building a relationship between the professional and the patient has benefits for recruitment as well as retention in trials [30]. In line with SLB, the reasoning behind this adopted recruitment strategy thus can be understood in terms of achieving recruitment success and using limited time resources optimally, and, from a functionalist perspective, providing care to patients who will benefit from participating. However, it could be argued that patients with a poor or non-existing relationship with the practice are possibly left behind. SLBs require compliance and cooperation from clients to fulfill their work tasks and control the client’s power of granting access to services and resources to the client [28]. GPs and staff rely on the patient’s cooperation and compliance to guarantee a more effective recruitment and trial process, and from the SLB perspective, the recruitment of patients who are already familiar in the practice can be seen as an act to ensure and achieve cooperation. 

#### The influence of digital assessment tools on discretion practices

In the SOFIA pilot study, record data from general practice and a SOFIA customization of the REDCap system was used for data collection and as a digital assessment tool assisting the GPs in performing the patient eligibility assessment. The digital solution was chosen to minimize discretion during the recruitment process. Building on Lipsky’s concepts, Bovens and Zouridis [31] argue that with the massive introduction of information technology in society, “street-level bureaucracy” has developed into “screen-level bureaucracy” where decision processes have been routinized by technology. This entails that discretion practices should become more systematized which could minimize the discretion performed by the ground-level worker [31]. On the other hand, other scholars argue that the impact of technology is determined by whom and how the technology is used [32], and studies investigating digital assessment tools in healthcare and social service settings argue that professionals perform technology workarounds because of heavy reporting burden or inflexible reporting templates [33, 34]. In the SOFIA study, the assessment of the SMI diagnosis was considered burdening and time-consuming and did in some cases lead to GPs working around the REDCap system when assessing eligibility. Furthermore, when variations exist in how SLBs perceive key concepts or categories applied in assessment tools it can influence the standardization between cases [34]. In the SOFIA pilot trial, the different understandings of what defines a *severe* mental illness that we identified in the interviews, and which has also been discussed in the literature [35], burdened the GPs in their assessment. 

### Strengths and limitations

The strength of this work lies in the solid empirical material that the analysis builds upon, which consists of quantitative process data, patient record data, and register data, alongside interviews with GPs and staff. Having the possibility of comparing included and eligible patients with a register sample and combining the analysis with the professionals’ perceptions provided us with different perspectives on the recruitment process.

The recruitment process data was registered by the GPs and staff themselves during the recruitment process which might introduce some challenges. There is a risk of underreporting baseline characteristics regarding comorbidities, previous use of GP services, reasons for exclusion, and patients’ reasons for declining participation. Although register data in Denmark is generally of high quality and validity[36], we only had access to diagnostic data from before the 1st of January 2019. The number of comorbidities might be higher in the SOFIA population as well as in the register population.

A dimension that might have provided further insight into the recruitment process, which we did not touch upon in this paper, is the patients’ perspectives. This aspect was outside the scope of the current study which primarily focused on the selection process taking place prior to the professionals contacting the patients. Still interviews with patients who accepted and declined participation could have developed our understanding of their considerations when being approached in the SOFIA trial. To our knowledge, patients' reasons for declining participation have primarily been investigated from the professionals’ viewpoint [10, 15]. However studies in other fields have found that patients decline participation in trials due to, i.e., not having the need for intervention, being too ill, having financial concerns, time constraints, and worrying about randomization [37–39]. Future studies could strive to address participation directly from the perspective of patients with SMI.

## Conclusion

In this study, we have shown that the recruitment strategy in the SOFIA pilot trial did not manage to include a representative sample of patients with SMI. The findings suggest that the recruitment strategy failed to recruit patients in more vulnerable or frail positions and might have excluded patients in need of intervention. These selection biases had two main drivers. The first driver was that GPs and staff mended eligibility criteria due to both (a) altruistic concerns for the patients and (b) considerations about maintaining work efficiency in the practice. The second driver was data-related challenges associated with the low quality of information extracted from the record data and use of a recruitment assessment tool which necessitated discretionary practices. Based on our findings, we suggest that the consequences of using professionals as recruiters and moreover using record data from general practice in combination with assessment tools are considered carefully when developing recruitment strategies for trials targeting patients with SMI in general practice.

## Data Availability

The datasets analyzed during the current study are not publicly available since they contain individual information on patients and practitioners. For inquiries on data requests please contact the corresponding author K. Tranberg (katrine.tranberg.jensen@sund.ku.dk).

## Appendix

**Table 4:** Interview guide patient recruitment—translated from Danish to English

**Theme**	**Content**	**Information and questions**
Briefing	Introduction of interviewer and study purpose	A brief introduction to the interviewer (KT) and what the study seeks to investigate.
	Data processing	Information for the participant on how we store data safely in the project, who has access to the interview, and how we anonymise names and personal information when sharing in presentations and publications.
	Payment	Their time was honored with a fee for every 10 min in the interview.
	Informed consent	Interlocuters were informed about the written consent, the meaning of the consent, that they were always able to withdraw their consent, whom to contact if they wanted to withdraw consent, and finally signed the consent form.
Introduction of the interlocuter	Work tasks, experience	I would like you to present yourself briefly: Will you tell me a bit about yourself, and your experience in general practice?
	Previous experience with patients with SMI	The SOFIA study is focused on patients with severe mental illnesses in general practice: Why did you/your practice choose to be a part of this study? Is the study of particular relevance to you?
Research question How was the patient recruitment performed in the SOFIA pilot trial and why?	Introduction to the recruitment process and distribution of work tasks	The next questions I have will be closely related to your recruitment of patients for the pilot study and will be aligned with the recruitment process elements that you performed in practice For starters, I would like to ask you how you divided the different work tasks during the recruitment? • How did you choose to divide the different tasks? • Who did what? • Did this division work well for you? • Did you change anything during the process—what and why? • How many from your practice participate in the study? • Which effect does this have?
	Extracting data from the GP record system	To identify patients with SMI in your system, the SOFIA project office has asked you to extract data from your record system on patients, their diseases, and previous contact to practice How did this process work for you? • How did you perform the data extraction? • Have you experienced any challenges in this regard? • Were you provided with the help you needed to perform this task? • In your experience—does the data you extracted match your expectations? • Why/why not? • In your opinion—do you consider this an appropriate method for identifying patients to the study?
	Patient exclusion process **(only to GPs)**	After you extracted patient data from your record systems, you were provided with a list of patients that potentially fulfilled the eligibility criteria from the SOFIA project office. Afterwards, you had to verify their diagnosis. Will you tell me how you approached that process? • Can you tell me how you reviewed the patients and why? • How much time did you spend on this part of the recruitment? • If you did not manage to review all patients on the list—why was that? • How did you choose to verify the patient’s diagnosis with SMI? • How did you experience this process? Was it easy or hard—can you give an example? • Did you find any diagnosis harder to verify than others? • How did you review the exclusion criteria? • Did you do this in a specific way—can you give an example? • Did you experience times, when you were in doubt and what did you do? • Would you say that there is anything that characterizes the patients that you included in the study/or excluded in the study? • How would you describe the patients you included in the study? • How would you describe the patients you excluded from the study? • Did you know all the patients on the list from before? • Did that affect how you included them? We know from research on the area, that many patients with SMI have co-occurring substance use. Is this something you experience when you review the patients and does this impact these patients’ ability to be included in the study?
	Contacting the patients	When you finished reviewing the patients, you were instructed to contact the patients you included in the study. Will you tell me how you performed this process and contacted the patients? • Who was primarily in charge of contacting the patients? • How did you contact them? • Was it possible to reach the patients? • Did you change the way you contacted the patients and why? I would like you to tell me about the conversations you had with the patients during the recruitment process—how did you experience this? • What did you tell them? • How did they react to your call? • What was it like for you? • Were there any challenges? • Did the patients ask any questions—and what did they ask you? • What worked particularly well during the conversations?
	Physical meeting in practice (collecting the informed consent)	**How did you experience the information meeting with the patient in practice?** • Where and how did you meet up? • What went well? • What was challenging? • Did the patient show? • Did they have any questions? • Did you feel well prepared for the conversations?
	Non-participants	I would like to ask you about the patients that declined to participate in the study. You have talked with them on the phone and/or if they attended the information meeting physically. Will you tell me about the patients that declined? • If you were to give your point of view—what characterizes the patients saying no? • Which reasons do the patients give for wanting to participate or not participate? • What do you think influences if the patient wants to participate? • What do you think impacts the project’s ability to recruit patients to the study—and why?
	Post-recruitment	Did the patients contact you after they agreed to participate—besides their regular contact and treatment? Why did they contact you and which questions did they have?
	Filling out the questionnaires	In the practice recruitment phase, some GPs and staff was concerned about the patients’ ability to fill out the questionnaires on quality of life in the study. Due to this, I would like to ask you if you have helped any of the patients fulfil the questionnaires—and how? spørgeskemaerne vedrørende livskvalitet enten i elektronisk—eller papirform. • How did you help them? • What did you help them with? • Which questions did they have? • Did they themselves have any concerns about filling out the questionnaires? • Did you experience any challenges in this regard?
	Registering process data on patient recruitment in REDCap	Connected to the recruitment, you were instructed to document the recruitment process in REDCap. I would like to hear your experiences and thoughts on using this system—will you tell me about that? • Did you use REDCap yourself? • If no—why was that? • Did you fill in all the information asked for? • What would make it easier for you to use the program?
	Info meetings during practice recruitment	Before you started recruiting patients, you had two meetings with the SOFIA project office—one concerning general information on participation and an additional meeting on the use of REDCap I would like to ask you what you gained from these meetings and whether there is anything you could have wanted to be different—in retrospect. • Did you feel ready for the task? • What do you think about having a GP inform you on the meetings instead of a project manager? • Did you contact the project office during the recruitment process for help? • Was the help you needed provided? • Moving forward—what could be improved?
	End	I am about to have runned thorugh all of the questions I had for you today regarding recruiting patients for the pilot study. Is there anything you would like to add, that we did not touch opon? Something you find relevant regarding the recruitment process or would have liked to be different?
Moving forward in the study	Comments regarding the project	Lastly, I want to ask you if you fell there is anything about the intervention/study that you feel is currently unclear? • Is there something that is not clear to you? • Something that will be of importance for you in the following process?
Debriefing	Final ending Turn of recorder “Off the record”	Thank you very much for taking the time for answering my questions. Now i have turned of the recorder—is there anything you would like to ask me off the record? Thank you again

## Abbreviations

GP/GPs: General practitioner/s
SMI: Severe mental illness
SUD: Substance use disorder
SLB/SLBs: Street-level bureaucrat/s
